# Legacy P and water quality perspective

**DOI:** 10.1002/jeq2.70285

**Published:** 2026-10-06

**Authors:** Sarah Stackpoole

**Affiliations:** ^1^ US Geological Survey Denver Colorado USA

**Abbreviation**
BMP: best management practice

## 1 PERSPECTIVE

My research has focused on national‐scale river phosphorus (P) water quality, which was motivated by a lack of apparent improvement despite considerable effort to manage or reduce nutrient inputs to freshwater ecosystems. In my work, I have defined legacy P as historical P surplus that has accumulated in a watershed over time, and it may be stored in agricultural soils, floodplains, or river sediments (Stackpoole et al., 2019; Stackpoole et al., 2021).

I focused on finding methods to quantify the relative contributions of contemporary as compared to legacy P sources on long‐term river loads and concentrations at a national scale, using a nutrient budget approach to estimate the magnitude of legacy P stored in watersheds, where the net sum of major P inputs and outputs is calculated at an annual timestep (Stackpoole et al., 2019; Sabo et al., 2021; Stackpoole et al., 2021). Inputs are fertilizer, manure, and wastewater, and the major output is crop P uptake and removal at harvest. A positive difference between inputs and outputs is considered a surplus and can be stored in the watershed and defined as legacy P. To better link the effects of historical legacy P to water quality conditions, I also developed a proxy estimate of legacy P contributions to river export (Stackpoole et al., 2019). I estimated mean watershed river loads and P balances over a 20‐year period. If the ratio of watershed mean river P export to the mean agricultural P balance was greater than one, I assumed that contemporary river P export in excess of the contemporary agricultural P balances was supported by legacy P sources.

A better understanding of the contribution of legacy and contemporary (current) sources to river P loads can be used to inform proper management actions and improve water quality. For example, in watersheds that had a P surplus and legacy P contributing to river P loads, a reduction in fertilizer and manure additions or increased drawdown of P in soils can be effective strategies to improve water quality (Zou et al., 2022; Mott et al., 2025). However, in some watersheds a reduction in P balances over time did not result in improved water quality conditions, or decreasing river P loads (Stackpoole et al., 2019). In fact, P balances were still positive, even though they had decreased over time, and using our proxy estimate for legacy P, I determined that historical P was likely not a significant source to the river P loads. I hypothesized that a change in management practices had occurred. For example, a riparian buffer strip, which for years had prevented the surplus P from reaching the river had been removed, leading to an eventual increase in river P loads. I did not have national‐scale datasets to characterize best management practice (BMP) changes across the watersheds over space or time. Improved documentation of various BMPs at regional to national scales, including vegetated buffer strips, cover crops, and other cultivation changes is necessary to explain some water quality patterns in response to contemporary and legacy P sources.

The P balances that I developed in my legacy P work were watershed based, but in the last 2 years, robust P balance datasets at the county, hydrologic unit code (Sabo, 2025), and 250‐m gridded scale (Byrnes, Chang, et al., 2026) have been developed. These advancements greatly enhance our ability to connect P balances with water quality (Brehob et al., 2025; Byrnes, Van Meter, et al., 2026). However, even with this increased spatial coverage, it is not possible to differentiate between the P stored in agricultural soils, floodplains, or river sediments, which has direct implications for management. Streambank loading can contribute P to rivers and streams, reducing water quality. But often this source of P is not documented, and is therefore misattributed (Margenot et al., 2023; Broerman et al., 2026). Robust spatial datasets of BMPs and documentation of legacy P that may have moved off agricultural fields and into streambanks provide metrics for achieving sustained improvements in river water quality and guiding effective nutrient management strategies.

## AUTHOR CONTRIBUTIONS

**Sarah Stackpoole**: Writing ‐ original draft; writing ‐ review and editing.

## CONFLICT OF INTEREST STATEMENT

The author declares no conflicts of interest.
